# Evaluation of multiple risk factors involved in the development of Diabetic Retinopathy

**DOI:** 10.12669/pjms.35.1.279

**Published:** 2019

**Authors:** Syeda Birjees Anwar, Naveed Asif, Syed Abid Hassan Naqvi, Sidra Malik

**Affiliations:** 1 Syeda Birjees Anwar, Department of Chemical Pathology, Armed Forces Institute of Pathology, Rawalpindi, Pakistan; 2 Naveed Asif, Department of Chemical Pathology, Armed Forces Institute of Pathology, Rawalpindi, Pakistan; 3 Syed Abid Hassan Naqvi, Armed Forces Institute of Ophthalmology, Rawalpindi, Pakistan; 4 Sidra Malik, Armed Forces Institute of Ophthalmology, Rawalpindi, Pakistan

**Keywords:** Diabetes mellitus, Blood Glucose Fasting (BGF), Glycosylated Hemoglobin (HbA1c), Diabetic retinopathy (DR)

## Abstract

**Objective:**

To determine the role of hypertension, hyperlipidemia, smoking and positive family history of diabetes and hypertension in the development of diabetic retinopathy.

**Methods:**

This prospective cohort study was conducted at the Department of Chemical Pathology, Armed Forces Institute of Pathology, Rawalpindi over 2 years period from June 2014 to June 2016. One hundred consecutive diabetic patients with no signs of diabetic retinopathy and good glycemic control (HbA1c<6.5%) were registered by non-probability convenient sampling after taking written informed consent. They were evaluated for hypertension, hyperlipidemia and smoking status. These patients were then followed 6 monthly for 2 years to look for the development of diabetic retinopathy.

**Results:**

The mean age of the patients was 50.72±9.29 years and there were 57 (57%) male and 43 (43%) female patients. Majority (82%) of the patients had NIDDM. The mean duration of diabetes was 8.31±6.83 years. 11% of the patients were smoker, 37% were hypertensive, 6% had hyperlipidaemia, 62% had family history of diabetes and 30% had family history of hypertension. At the end of follow-up, 9 (9.0%) patients had diabetic retinopathy. The frequency of diabetic retinopathy increased with increasing age of the patient; however, the difference was statistically insignificant. A comparatively higher frequency of diabetic retinopathy was also seen in patients with IDDM and those with positive family history of diabetes and hypertension yet again, the difference was statistically insignificant. Also, no significant difference was noted among male and female genders and smokers vs. non-smoker. However, the frequency of diabetic retinopathy increased significantly with increasing duration of diabetes. It was also higher among those with hypertension and hyperlipidemia.

**Conclusion:**

Higher patient age (≥50 years), increasing duration of diabetes (≥20 years), insulin dependent diabetes mellitus, hypertension, hyperlipidemia, and positive family history of diabetes and hypertension were found to be associated with increased frequency of diabetic retinopathy.

## INTRODUCTION

Diabetes is a metabolic syndrome resulting from hyperglycemia due to decreased indigenous insulin, decreased sensitivity to circulating insulin or both.^1^ The estimated world population with diabetes was 366 million in 2011 and is estimated to grow to 552 million in the year 2030. Disease burden is relatively higher in developing countries which comprise around 80% of the world diabetic population.^2^ According to WHO estimate, Pakistan had 6.9 million diabetic patients in 2007; a figure which is estimated to become 11.5 million in 2025.^3^

Diabetes is a leading cause of preventable blindness in adults aged between 20-75 years.^4^ Among the ocular complications of diabetes, diabetic retinopathy is the most frequent and it is responsible for 12% of all new cases of blindness every year.^4^,^5^ Among patients of type-I and type-II diabetes for more than 20 years, the prevalence of diabetic retinopathy has been reported to be 95% and 60%, respectively.^6^ However, the prevalence varies depending upon the population and the age group being studied.^4^-^6^

Existing research has identified a number of factors attributable for the development of diabetic retinopathy including but not limited to duration of diabetes along with glycemic control, systemic hypertension, hyperlipidemia, obesity and positive family history of diabetes.^7^-^12^ Prevention and treatment of these attributing factors is as much important as good glycemic control to avoid diabetic retinopathy.^7^,^9^,^11^,^12^

Although these risk factors have already been studied in many epidemiological and clinical trials, yet there were considerable differences in the consistency, pattern, and strength of these factors among these studies. As no such local published material could be found, the purpose of the current study was to determine the role of hypertension, hyperlipidemia, smoking and positive family history of diabetes and hypertension in the development of diabetic retinopathy with the hope that the results of this study would help in early identification of high risk diabetic patients allowing timely intervention to reduce the burden of diabetic retinopathy.

## METHODS

This was a cohort study conducted at the Department of Chemical Pathology, Armed forces institute of Pathology, Rawalpindi over 2 years period from June 2014 through June 2016. One hundred consecutive diabetic patients reporting at Ophthalmology outdoor were screened on their first visit for presence or absence of diabetic retinopathy. Those patients with no signs of diabetic retinopathy and good glycemic control (HbA1c<6.5%) were registered after taking written informed consent. They were evaluated for hypertension, hyperlipidemia and smoking status. These patients were then followed 6 monthly for 2 years to look for the occurrence of diabetic retinopathy. Patients of ischemic heart disease, chronic liver and kidney diseases were excluded. Same consultant ophthalmologist examined all the patients on every follow-up to eliminate bias.

## RESULTS

The mean age of the patients was 50.72±9.29 years and there were 57 (57%) male and 43 (43%) female patients. Majority (82%) of patients had NIDDM. The mean duration of diabetes was 8.31±6.83 years. 11% of the patients were smoker, 37% were hypertensive, 6% had hyperlipidemia, 62% had family history of diabetes and 30% had family history of hypertension. These findings have been summarized in Table-I.

**Table-I:** Baseline Characteristics of the Study Cohort.

Characteristics	Participants n=100
Age	50.72±9.29 years
*Gender*	
• Male	57 (57.0%)
• Female	43 (43.0%)
*Type of diabetes*	
• IDDM	18 (18.0%)
• NIDDM	82 (82.0%)
Duration of diabetes	8.31±6.83 years
*Comorbid*	
• Hypertension	37 (37.0%)
• Hyperlipidemia	6 (6.0%)
• Smoker	11 (11.0%)
• Family history of diabetes	62 (62.0%)
• Family history of hypertension	30 (30.0%)

At the end of follow-up, 9 (9.0%) patients had diabetic retinopathy. The frequency of diabetic retinopathy increased with increasing age of the patient; 30-40 vs. 41-50 vs. 51-60 years (0.0% vs. 4.5% vs. 14.3%; p=0.099; OR=0.286, 95% CI: 0.03-2.43) however, the difference was statistically insignificant. A comparatively higher frequency of diabetic retinopathy was also seen in patients with IDDM (16.7% vs. 7.3%; p=0.209; OR=0.40, 95% CI: 0.09-1.76) and those with positive family history of diabetes (12.9% vs. 2.6%; p=0.081; OR=5.48, 95% CI: 0.66-45.69) and hypertension (13.3% vs. 7.1%; p=0.322; OR=2.00, 95% CI: 0.50-8.04) yet again, the difference was statistically insignificant. Also no significant difference was noted among male and female genders (8.8% vs. 9.3%; p=0.927; OR=0.938, 95% CI: 0.24-3.72) and smokers vs. non-smokers (9.1% vs. 9.0%; p=0.991; OR=1.013, 95% CI: 0.11-8.96).

However, the frequency of diabetic retinopathy increased significantly with increasing duration of diabetes; 0-10 vs. 11-20 vs. 21-30 years (5.2% vs. 14.3% vs. 25.0%; p=0.037; OR=2.00, 95% CI: 0.18-22.06). It was also higher among those with hypertension (18.9% vs. 3.2%; p=0.008; OR=7.12, 95% CI: 1.39-36.36) and hyperlipidemia (33.3% vs. 7.4%; p=0.032; OR=6.21, 95% CI: 0.96-40.07) as shown in Table-II.

**Table-II:** Patients characteristics including risk factors

Variable	Diabetic Retinopathy n (%)	P value	OR	95% CI
Overall	9 (9.0%)	-	-	-
*Age Group*				
• 30-40 years	0/22 (0.0%)	0.099		
• 41-50 years	1/22 (4.5%)		0.000	0.000
• 51-60 years	8/56 (14.3%)		0.286	0.03 – 2.43
*Gender*				
• Male	5/57 (8.8%)	0.927	0.938	0.24 – 3.72
• Female	4/43 (9.3%)
*Duration of Diabetes*				
• 0-10 years	4/77 (5.2%)	0.037*		
• 11-20 years	1/7 (14.3%)		0.329	0.03 – 3.43
• 21-30 years	4/16 (25.0%)		2.000	0.18 – 22.06
*Type of Diabetes*				
• NIDDM	6/82 (7.3%)	0.209	0.395	0.09 – 1.76
• IDDM	3/18 (16.7%)
*Hypertension*				
• No	2/63 (3.2%)	0.008*	7.117	1.39 – 36.36
• Yes	7/37 (18.9%)
*Hyperlipidaemia*				
• No	7/94 (7.4%)	0.032*	6.214	0.96 – 40.07
• Yes	2/6 (33.3%)
*Smoking*				
• No	8/89 (9.0%)	0.991	1.013	0.11 – 8.96
• Yes	1/11 (9.1%)
*Family History of Diabetes*				
• No	1/38 (2.6%)	0.081	5.481	0.66 – 45.69
• Yes	8/62 (12.9%)
*Family History of Hypertension*				
• No	5/70 (7.1%)	0.322	2.000	0.50 – 8.04
• Yes	4/30 (13.3%)			

### Statistical analysis

Inferential statistics were applied by using SPSS version 21. Mean and standard deviation were calculated for quantitative data. Categorical variables were presented by frequency and percentages. Association was between categorical variables and was calculated by using Chi Square test. The p-value ≤ 0.05 was considered significant.

## DISCUSSION

In the present study, the age of the patients ranged from 30 years to 60 years with a mean of 50.72±9.29. Cheng et al. in 2009 (55.9±0.61 years)^13^ and Memon et al. in 2012 (55.3±8.9 years)^14^ observed similar mean age among diabetic patients in American and Pakistani populations respectively. There were 57 (57%) male and 43 (43%) female patients in the present study. Chuhan et al. also observed male predominance (63.12% vs. 36.88%) among diabetic patients at Kashmir.^15^ Memon et al. (1:1.2)^14^ and Ahmed et al. (1:1.3)^16^ however observed slight female predominance at Karachi and Abbottabad, Pakistan respectively.

Majority (82%) of patients had NIDDM while only 18% had IDDM. Rahman et al. in 2011 observed a similar frequency of IDDM (16%) among diabetic patients presenting at a teaching hospital in Abbottabad, Pakistan.^17^ Ahmed et al. however observed quiet lower frequency of 5.14% in Abbottabad, Pakistan.^16^ The duration of diabetes ranged from 0 to 30 years with a mean of 8.31±6.83 years. A similar mean duration of diabetes was reported by Ahmedani et al. in 2005 (8.88±5.21 years) among patients of diabetes evaluated for Microalbuminuria^18^ 11% of the patients were smoker, 37% were hypertensive, 6% had hyperlipidemia, 62% had family history of diabetes and 30% had family history of hypertension. A much higher frequency of hypertension was previously observed by Rahman et al. in 2011 (53.5%)^17^ and Ahmedani et al. in 2005 (55.9%).^18^ Ahmedani et al. also reported variable frequency of smoking (29.5%), family history of diabetes (44.6%) and hypertension (5%) among diabetic patients.^18^

Upon final follow-up visit, 9 patients had diabetic retinopathy. This gave an overall frequency of diabetic retinopathy to be 9% among diabetic patients irrespective of diabetes control status in the present study. A similar frequency of diabetic retinopathy (10%) was observed by Shaikh et al. in 2010 among diabetic patients after 10 years of diagnosis while the mean duration of diabetes in our study was 8 years.^19^ Wahab et al. in 2008 (15%)^20^ and Mahar et al. in 2010 (27.43%)^21^ reported relatively higher frequency of DR in local population. Among other populations Cheng et al. in US (11%)^13^, Wong et al. in Australia (11.5%)^22^, Nathan (12.6%)^23^ and Agarwal et al. (11.7%)^24^ in India and Abdollahi et al. in Iran (13.8%)^25^ reported similar frequency of diabetic retinopathy. A much higher frequency of 49.94% was reported by Khanzada et al. in 2011.^26^ A possible explanation for this much higher frequency can be the prolonged duration of diabetes among the participants of that study (mean duration=13±4.5 years).^26^ This variation can be explained by socioeconomic (availability and affordability of treatment) and educational differences (interest and compliance) in populations and the selection bias among researchers.^20^

The frequency of diabetic retinopathy increased with increasing age of the patient (OR=0.286, 95% CI: 0.03-2.43; p=0.09). Wong et al. in 2008 also observed insignificant difference in the frequency of diabetic retinopathy with age (OR=0.93, 95% CI:0.79–1.09; p=0.38).^22^ Hu et al. in 2015 (OR=2.08, 95% CI:1.52-2.83; p<0.001)^11^ and Raman et al. in 2015 (OR=2.19, 95% CI:1.29-3.73; p=0.004)^12^ however observed significantly increased risk of diabetic retinopathy with increasing age. We didn’t observe any significant difference among male and female genders (8.8% vs. 9.3%; p=0.927; OR=0.938, 95% CI: 0.24-3.72). Our results are similar to those of Hu et al. who also didn’t observe any significant difference with gender (OR=0.95, 95% CI: 0.74-1.22; p=0.662).^11^ Raman et al. in 2015 (OR=1.66, 95% CI:1.14-2.42; p=0.009)^12^ and Chatziralli et al. in 2010 (OR=3.57, 95% CI:1.67-7.62; p=0.001)^10^ however reported increased risk of diabetic retinopathy in males.

A comparatively higher frequency of diabetic retinopathy was also seen in patients with IDDM (16.7% vs. 7.3%; p=0.209; OR=0.40, 95% CI: 0.09-1.76) and those with positive family history of diabetes (12.9% vs. 2.6%; p=0.081; OR=5.48, 95% CI: 0.66-45.69) and hypertension (13.3% vs. 7.1%; p=0.322; OR=2.00, 95% CI: 0.50-8.04. Hu et al. (OR=1.52, 95% CI:1.20-1.92; p=0.001)^11^ also observed similar risk with positive family history of diabetes. The frequency of diabetic retinopathy increased significantly with increasing duration of diabetes; 0-10 vs. 11-20 vs. 21-30 years (5.2% vs. 14.3% vs. 25.0%; p=0.037; OR=2.00, 95% CI: 0.18-22.06).

It was also higher among those with hypertension (18.9% vs. 3.2%; p=0.008; OR=7.12, 95% CI:1.39-36.36) and hyperlipidemia (33.3% vs. 7.4%; p=0.032; OR=6.21, 95% CI:0.96-40.07). A similar increased risk of diabetic retinopathy has been reported in association with hypertension by Chatziralli et al. (OR=4.49, 95% CI:1.15-17.49; p=0.030)^10^. While Insignificant difference was observed by Wong et al. in relation to hyperlipidemia (OR=0.88, 95% CI: 0.65–1.19; p=0.39).^22^

The results of the present study are similar to those of previously published studies in this regard among other populations with minor dissimilarities which can be due to population differences. The present study is first of its kind in local population and has identified some very important modifiable risk factors among diabetic patients, timely identification and management of which can help in reducing the development of diabetic retinopathy.

## CONCLUSION

Higher patient age (≥50 years), increasing duration of diabetes (≥20 years), insulin dependent diabetes mellitus, hypertension, hyperlipidemia, and positive family history of diabetes and hypertension were found to be associated with increased frequency of diabetic retinopathy. Thus, these factors should be taken into account for control along with glycemic control to decrease the risk of diabetic retinopathy in practice.

### Authors’ Contribution

***SBA*** conceived, designed and did statistical analysis & editing of manuscript.

***SAHN and SM*** did data collection and manuscript writing.

***NA*** did review and final approval of manuscript.

## References

[1] Olokoba AB, Obateru OA, Olokoba LB (2012). Type 2 diabetes mellitus:a review of current trends. Oman Med J.

[2] (2017). International Diabetes Federation. IDF Diabetes Atlas.

[3] The Nation WHO ranks Pakistan 7th on diabetes prevalence lists.

[4] Manaviat MR, Rashidi M, Afkhami-Ardekani M, Shoja MR (2008). Prevalence of dry eye syndrome and diabetic retinopathy in type 2 diabetic patients. BMC Ophthalmol.

[5] da Silva Corrêa ZM, Freitas AM, Marcon IM (2003). Risk factors related to the severity of diabetic retinopathy. Arq Bras Oftalmol.

[6] Garg S, Davis RM (2009). Diabetic Retinopathy Screening Update. Clin Diab.

[7] Yau JW, Rogers SL, Kawasaki R, Lamoureux EL, Kowalski JW, Bek T (2012). Global prevalence and major risk factors of diabetic retinopathy. Diabetes Care.

[8] Penman A, Hancock H, Papavasileiou E, James M, Idowu O, Riche DM (2016). Risk Factors for Proliferative Diabetic Retinopathy in African Americans with Type 2 Diabetes. Ophthalmic Epidemiol.

[9] Xu J, Wei WB, Yuan MX, Yuan SY, Wan G, Zheng YY (2012). Prevalence and risk factors for diabetic retinopathy:the Beijing Communities Diabetes Study 6. Retina.

[10] Chatziralli IP, Sergentanis TN, Keryttopoulos P, Vatkalis N, Agorastos A, Papazisis L (2010). Risk factors associated with diabetic retinopathy in patients with diabetes mellitus type 2. BMC Res Notes.

[11] Hu Y, Teng W, Liu L, Chen K, Liu L, Hua R (2015). Prevalence and risk factors of diabetes and diabetic retinopathy in Liaoning province, China:a population-based cross-sectional study. PLoS One.

[12] Raman R, Ganesan S, Pal SS, Prevalence and risk factors for diabetic retinopathy in rural India (2014). Sankara Nethralaya Diabetic Retinopathy Epidemiology and Molecular Genetic Study III (SN-DREAMS III), report no 2. BMJ Open Diabetes Res Care.

[13] Cheng YJ, Gregg EW, Geiss LS, Imperatore G, Williams DE, Zhang X (2009). Association of A1C and fasting glucose levels with diabetic retinopathy prevalence in the U.S. population:Implications for diabetes diagnostic thresholds. Diabetes Care.

[14] Memon WA, Jadoon Z, Qidwai U, Naz S, Dawar S, Hasan T (2012). Prevalence of diabetic retinopathy in patients of age group 30 years and above attending multicenter diabetic clinics in Karachi. Pak J Ophthalmol.

[15] Chuhan R, Dahri GM, Murad S, Raza H, Akram S (2010). Prevalence and incidence of diabetes mellitus in rural areas of Sindh province of Pakistan. Pak J Med Health Sci.

[16] Ahmed K, Muhamamd Z, Qayum I (2009). Prevalence of cutaneous manifestations of diabetes mellitus. J Ayub Med Coll Abbott.

[17] Rahman S, Nawaz R, Khan GJ, Aamir AH (2011). Frequency of diabetic retinopathy in hypertensive diabetic patients in a tertiary care hospital of Peshawar, Pakistan. J Ayub Med Coll Abbott.

[18] Ahmedani MY, Iqbal Hydrie MZ, Iqbal A, Gul A, Mirza WB, Basit A (2005). Prevalence of microalbuminuria in type 2 diabetic patients in Karachi:Pakistan a multi-center study. J Pak Med Assoc.

[19] Shaikh MA, Gillani S, Dur-e-Yakta (2010). Frequency of diabetic retinopathy in patients after ten years of diagnosis of type 2 diabetes mellitus. J Ayub Med Coll Abbott.

[20] Wahab S, Mahmood N, Shaikh Z, Kazmi WH (2008). Frequency of retinopathy in newly diagnosed type 2 diabetes patients. J Pak Med Assoc.

[21] Mahar PS, Awan MZ, Manzar N, Memon MS (2010). Prevalence of type-ii diabetes mellitus and diabetic retinopathy:the Gaddap Study. J Coll Physicians Surg Pak.

[22] Wong TY, Liew G, Tapp RJ, Schmidt MI, Wang JJ, Mitchell P (2008). Relation between fasting glucose and retinopathy for diagnosis of diabetes:three population-based cross-sectional studies. Lancet.

[23] Diabetes Prevention Program Research Group (2007). The prevalence of retinopathy in impaired glucose tolerance and recent-onset diabetes in the Diabetes Prevention Program. Diabetic Med.

[24] Agarwal S, Raman R, Kumari RP, Deshmukh H, Paul PG, Gnanamoorthy P (2006). Diabetic retinopathy in type II diabetics detected by targeted screening versus newly diagnosed in general practice. Ann Acad Med Singapore.

[25] Abdollahi A, Malekmadani MH, Mansoori MR, Bostak A, Abbaszadeh MR, Mirshahi A (2006). Prevalence of diabetic retinopathy inpatients with newly diagnosed type II diabetes mellitus. Acta Medica Iranica.

[26] Khanzada MA, Narsani AK, Shaikh F, Jatoi SM (2011). Frequency and types of diabetic retinopathy in type II diabetes;a hospital base study. J Liaquat Uni Med Health Sci.

